# A methodological exploration to study 2D arm kinematics in Ophiuroidea (Echinodermata)

**DOI:** 10.1186/s12983-023-00495-y

**Published:** 2023-04-21

**Authors:** Mona Goharimanesh, Sabine Stöhr, Fereshteh Ghassemzadeh, Omid Mirshamsi, Dominique Adriaens

**Affiliations:** 1 Department of Biology, Ferdowsi University of Mashhad, Mashhad, Iran; 2 Research Group Evolutionary Morphology of Vertebrates, Ghent University, Ghent, Belgium; 3 Department of Zoology, Swedish Museum of Natural History, Stockholm, Sweden

**Keywords:** Arm movement, Brittle star, Image processing, Locomotion

## Abstract

Brittle stars, unlike most other echinoderms, do not use their small tube feet for locomotion but instead use their flexible arms to produce a rowing or reverse rowing movement. They are among the fastest-moving echinoderms with the ability of complex locomotory behaviors. Considering the high species diversity and variability in morphotypes, a proper understanding of intra- and interspecies variation in arm flexibility and movement is lacking. This study focuses on the exploration of the methods to investigate the variability in brittle star locomotion and individual arm use. We performed a two-dimensional (2D) image processing on horizontal movement only. The result indicated that sinuosity, disc displacement and arm angle are important parameters to interpret ophiuroid locomotion. A dedicated Python script to calculate the studied movement parameters and visualize the results applicable to all 5-armed brittle stars was developed. These results can serve as the basis for further research in robotics inspired by brittle star locomotion.

**Supplementary Information:**

The online version contains supplementary material available at 10.1186/s12983-023-00495-y.

## Introduction

Brittle stars (Ophiuroidea) bear five distinctive arms, attached to a central disc containing the internal organs, in a pentaradial symmetry [1]. An internal skeleton of ossicles (called arm vertebrae) supports the arms. These vertebrae articulate proximally and distally, and are controlled by a set of muscles [2, 3]. Generally, ophiuroid motion includes ‘movement’ (displacement of the disc or arm from its original position) and ‘locomotion’ (displacement of the animal from one place to another). Ophiuroids, unlike asteroids (sea stars), do not use their numerous, small tube feet for locomotion but rely on their flexible arms to produce a rowing or reverse rowing movement [2, 4].’Rowing’ refers to the arms of the brittle star exerting a force on the substrate to push itself forward. During this type of movement, the leading arm is oriented in the direction of movement, whereas in ‘reverse rowing’ it is in the opposite direction [5]. In the absence of a head/tail axis, ophiuroids change the moving direction not by turning their disc, but by switching the leading arm. This fact allows them to easily react to stimuli provided from a different side [6]. They execute coordinated movements and move in a plane perpendicular to their central (ventral–dorsal) axis, despite a lack of a centralized control [4, 5, 7]. Brittle stars are among the fastest-moving echinoderms with the ability of complex locomotory behaviors, even when one or more arms are lost [8, 9]. This makes them interesting model organisms in robotics [2, 8, 10–12]. Several studies focused on brittle star motion and its control by a nervous ring [6, 13, 14]. However, considering the high species diversity and variability in morphotypes, a proper understanding of intra- and interspecies variation in arm flexibility and movement is still lacking. Developing kinematic analyses and further behavioral experiments in ophiuroid motion can assist in understanding how a decentralized control setup coordinates brittle star locomotion.

This study focuses on methods to properly characterize (in 2D) arm kinematics during brittle star locomotion, and to summarize the complexity and variability in individual arm use into a visually comprehensible manner. In addition, we hypothesized that the reverse rowing kinematic differs from a forward rowing one, with respect to studied arm motion. In this study, we studied the locomotion kinematics in three species: *Ophiocoma scolopendrina* (Lamarck, 1816) (Ophiocomidae), *Ophiolepis superba* H.L. Clark, 1915 (Ophiolepididae) and *Macrophiothrix hirsuta* (Müller & Troschel, 1842) (Ophiotrichidae). *Ophiolepis superba* and *M. hirsuta* belong to the same order Amphilepidida, but *O. scolopendrina* belongs to Ophiacanthida. According to our personal observations, *O. scolopendrina* lives under rocks and coral rubble in shallow water of the upper intertidal zone. *Macrophiothrix hirsuta*, a species with long arms occupies intertidal sand/mudflats, space/crevices between rocks/pieces of coral rubble and underlying substrate and lives among sponges [15]. In contrast, *O. superba* belongs to the subtidal zone, lower littoral and deeper, living among corals and hidden under rocks [16]. The three species were selected because they differed from each other in one or more morphological traits. The general external and internal morphological differences of these three species were summarized in Supp. File 1, based on Goharimanesh et al. [17]. Recent studies on vertebral morphology in *M. hirsuta*, *O. scolopendrina* [15], and *O. superba* [18] indicated that they all have vertebrae with a zygospondylous articular structure. However, *M. hirsuta* has very long arms (> 4 × disc diameter), with vertebrae with a comb-shaped zygospondylous articulation and extended keel [15, 17, 19]. *Ophiocoma scolopendrina* and *O. superba* have a medium arm length (3—4 × disc diameter) with a universal zygospondylous articulation and short-keeled vertebrae [15, 17, 19, 20]. Goharimanesh et al. [15] reported that the vertebrae in *O. scolopendrina* are larger than in *M. hirsuta*, with a longer process at the dorsal projection. Also, the muscular fossae are deeper on both proximal and distal faces of the vertebrae, which suggests the insertion of larger muscles. However, the relation between the morphological structures and locomotion behavior has not been properly investigated in brittle stars yet. Thus, we chose the three species that are phylogenetically and morphologically very distinct, as case studies for a methodological exploration of new variables and ways to summarize arm kinematics graphically. We also aimed to investigate whether this method captures variability in kinematics properly.

We performed a two-dimensional (2D) image processing on horizontal movement only. A dedicated Python script to calculate the studied movement parameters and visualize the results was developed and provided as supplementary information Additional file. 1 (https://github.com/mgm1001/Locomotion_Ophiuroidea.git). The new kinematic parameters and the scripts can be implemented to apply to all ophiuroid species and therefore illuminate the relation between structure and function.

## Material and methods

### Specimens

Five intact individuals of the banded brittle star *Ophiolepis superba* H.L. Clark, 1915 were used. This species has distinct colors on the segments (a banded pattern), making it suitable for accurately tracking their locomotion. The specimens of *O. superba* were purchased from the pet trade (Poission d’Or, https://www.poisson-or.com/) and kept in a laboratory aquarium (120 × 60 × 50 cm) at the research group Evolutionary Morphology of Vertebrates at Ghent University. The aquarium was filled with artificial seawater at 22–28 °C, pH 8.0–8.4, specific gravity (relative density) 1.023–1.025, and salinity of 3.2–3.3% (Synthetic marine salt from Aquaforest.eu). The brittle stars were fed weekly with small crustaceans (*Mysis* and *Artemia*). The specimens of *O*. *superba* were on average 1.57 ± 0.05 cm in disc diameter and 5.39 ± 0.56 cm in arm length. In addition, six individuals of *Ophiocoma scolopendrina* (Lamarck, 1816) (including four juvenile individuals of miniature size) and six individuals of *Macrophiothrix hirsuta* (Müller & Troschel, 1842) were added to the analysis by recording their movement immediately on site after having collected them alive during December 2017–March 2018 in Dayyer, Nayband bay, Tis, and Chabahar Marine University (Iran) [for details see Goharimanesh et al. [19]]. Following the recording, the animals were fixed in neutralized buffered formalin for further anatomical study [15].

### Experimental setup

For the controlled experiment, each specimen of *Ophiolepis superba* was placed in a flat plastic case (55.5 × 36 × 21 cm) filled with artificial seawater from the aquarium they were housed in. During video recording, illumination was kept constant and homogenous, to avoid a light gradient. Locomotion was recorded in dorsal view using a JVC HD Everio GZ-GX1 digital camera (resolution 1920 × 1080 pixels, at 300 fps). The locomotion of *Ophiocoma scolopendrina* and *Macrophiothrix hirsuta* was recorded at each collecting site (see above) in a styrofoam case filled with local seawater, using a Nikon FullHD S6400 digital camera (1920 × 1080, at 30 fps). Videos were saved in MP4 format. For each experiment, the specimens were left in the center of the case and allowed to move freely. We repeated the experiment five times for each specimen and each time the specimen was rotated clockwise to randomize the spatial orientation of the arms [13]. One video per individual was then selected in which either rowing or reverse rowing in mainly one direction was recorded. The species we studied naturally avoid light and open spaces, therefore when placed in the center of the tank, they immediately moved to a corner, during which the movement was recorded.

### Locomotion analysis

**Fig. 1:**
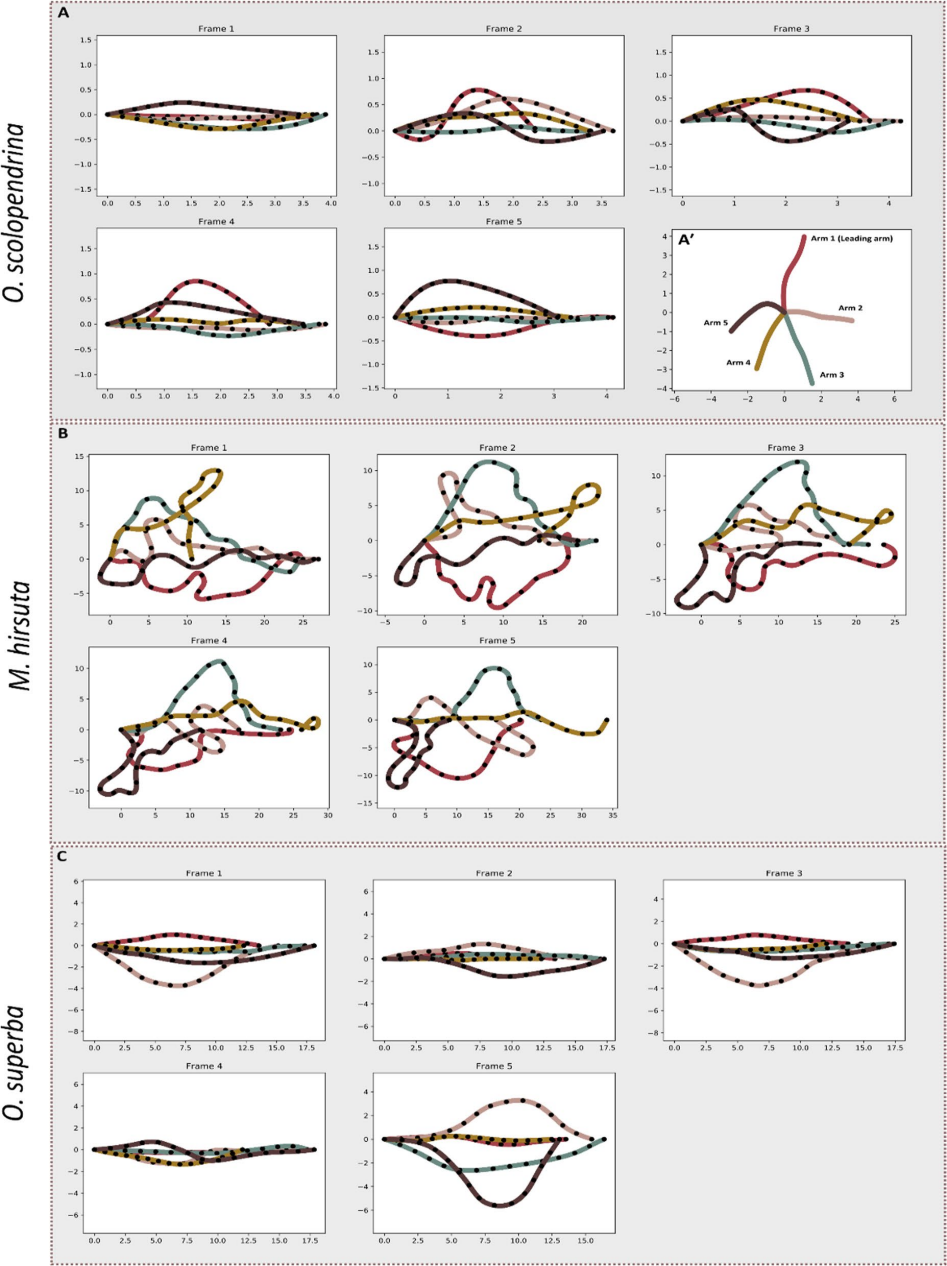
Superimposed and aligned curve of the arms in frames 1–5 within one trial for *O. scolopendrina* (**A**), *M. hirsuta* (**B**) and *O. superba* (**C**) after axes transformation and axes rotation. The lower right corner of plot A shows an arm configuration before axis transformation (A′). The filled circles on the arms show equidistant landmarks. The arm numbers in each plot follows A′. The axes imply the X and Y coordinates in the transformed Cartesian coordinate system in cm

For the kinematic analysis, six videos from each species and one additional of reverse rowing in *O. superba* were chosen for image processing (19 videos in total). From each cropped video, a full cycle of arm movement (from stance to swing positions of both arms 2 and 5) was split into four equal intervals, yielding five frames that were saved in jpg format. These images were then analyzed using Kappa, a Fiji plugin for curvature analysis [21]. B-spline curves were drawn by the control point tool starting from the most proximal part of each arm to the most distal part, following the arm midline. Individual arm movement was quantified relative to the direction of motion of the central disc for each cycle of movement. The leading arm was oriented parallel to the direction of motion and was named arm 1, with the following arms named in a clockwise manner (Fig. 1A′). Tracking the arm trajectories resulted in X, Y coordinates of the points describing each curve (arm), which were extracted in.csv format for input in the Python script (v.3) for further analysis.

To obtain coordinates that reflect arm kinematics in a comparable manner, several steps of data standardization were performed. For axes transformation, the coordinates of the most proximal point of every arm were subtracted from those of every arm point. Subsequently, the proximal part of all arms was superimposed in the zero position of a Cartesian coordinate system. For the axes rotation, we defined several conditional arguments (Axis rotation in https://github.com/mgm1001/Locomotion_Ophiuroidea.git) for the required angle rotation of each arm to be individually aligned to the X-axis and to calculate the new coordinates in the new XY Cartesian coordinate system. In this regard, individual arms were aligned by positioning the proximal and distal points of the arms on the X-axis (Fig. 1).

**Fig. 2:**
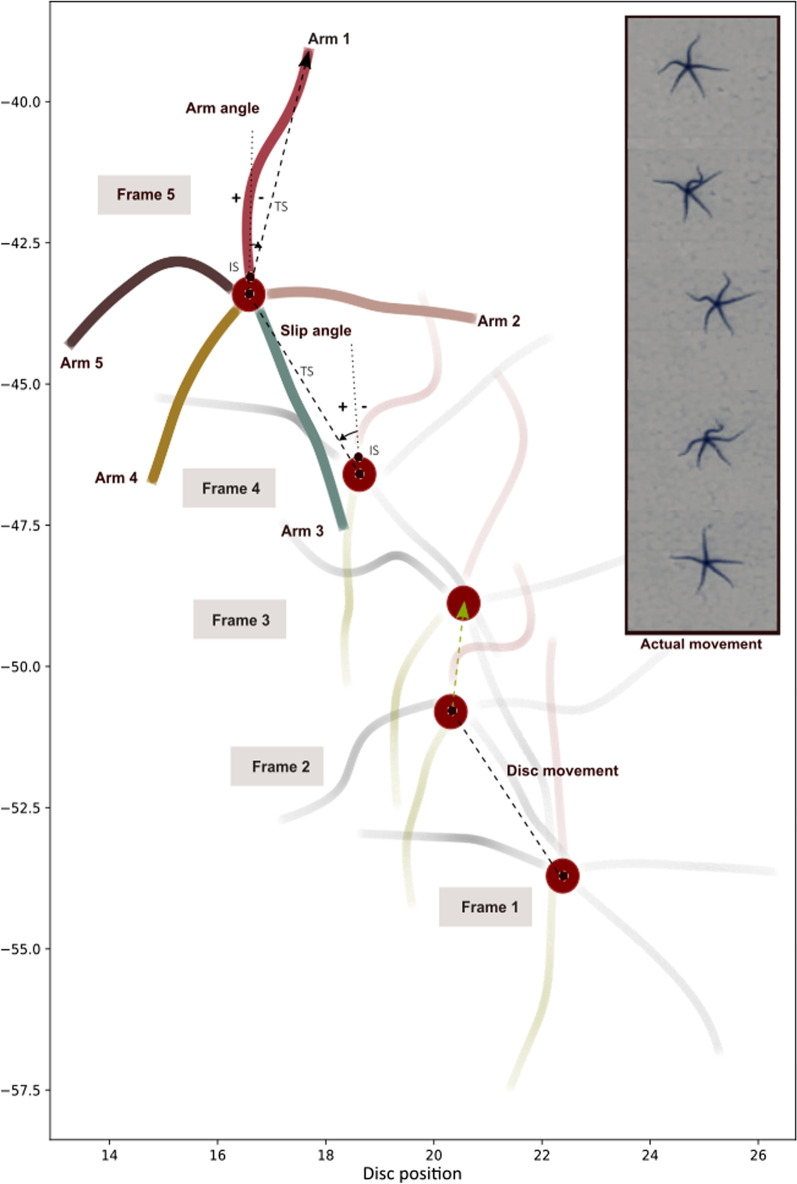
Visualisation of the actual movement in one trial of *O. scolopendrina* within time frames 1–5, indicating the variables disc movement, arm angle, and slip angle. IS, Initital side of the angle; TS, Terminal side of the angle. The axes imply the X and Y coordinates in the transformed Cartesian coordinate system in cm. The figure implies a full cycle of arm movement which was split into four equal intervals, yielding five frames

To do the kinematic analyses we examined four variables, namely the sinuosity index, slip angle, arm angle and disc displacement. Sinuosity is the ability to curve, which for ophiuroids implies that larger values indicate increased arm flexibility. Using this index, variable arm bending capacities in a horizontal plane could be quantified with a single numerical value. The second variable, slip angle or sideslip angle, is a term in vehicle mechanics that defines the angle between direction that a wheel is pointing and the direction it is travelling (Leucht [22]). We used this by calculating the angle between the direction in which the proximal part of each arm is pointing with respect to the central disc and the direction in which the animal is moving (quantified as the direction of the moving central disc). This angle helps us to infer the locomotion direction and disc rotation of the animal (Fig. 2). In addition to slip angle, we included arm angle as a variable, using a slightly modified approach used by Astley [5] and Wakita et al. [14]. Unlike Wakita et al. [14], we used the whole curvature and calculated arm angle from the distal point, rather than the middle arm point, to the disc center (as terminal side of the angle). The whole curvature is needed to observe the complete arm behavior and to compare with two other variables, i.e., sinuosity and slip angle that also mainly stand for whole arm curvature. It is worthwhile mentioning that, when an arm has the lowest sinuosity (close to 1), it shows an accurate estimation of overall arm angle because probable erratic movements in the distal part, especially in animals with very long arms, were removed, whereas, in high-sinuosity cases the distal part could affect the arm angle. In addition, we used the arm base (where it attaches to the disc) as the initial side of the angle similar to Wakita et al. [14], but different from Astley [5], who only used the disc center to the distal point of each arm. By using the line connecting this arm base to the disc center, we could record whether angular movement was in a positive or negative direction along the initial side of the angle, which is not possible with the approach used by Astley [5]. A positive angle is measured in a counterclockwise direction from the initial side of the angle to the terminal side, and negative angle is in a clockwise direction.

To calculate the ‘sinuosity’, the number of points used to track each arm was reduced to 15 equidistant landmarks on each arm (based on the evaluation of point number on the curves in one trial per species), using the *interp1d ()* function of SciPy in Python. Next, by using the Pythagorean theorem, the arm ‘length’ was calculated by the sum of the distances between the consecutive 15 points and the arm ‘distance’ was calculated by the distance between the most proximal and most distal point of the arm (Variable_1 in https://github.com/mgm1001/Locomotion_Ophiuroidea.git). Finally, sinuosity is expressed as the ratio of the arm length versus the arm distance (larger values represent arms more heavily flexed).

The ‘arm angle’ (Fig. 2) was obtained by calculating the angle between the line connecting the arm's distal point to the disc center (terminal side of the angle) and the line between the arm’s proximal point to the disc center (initial side), which is the disc radius. Coordinates of the disc center were calculated by averaging the coordinates of the most proximal point of the five arms. To transform the disc center to the zero position of a new Cartesian coordinate system and calculate the arm angle, we subtracted the coordinates of the disc center from the coordinates of the most proximal and distal points of the arm, as well as the disc center point within each frame. Angle direction was standardized for positive and negative angle changes for each arm, with separate conditions for either of the four axis quadrants where each arm is located (Variable_2 in https://github.com/mgm1001/Locomotion_Ophiuroidea.git). A positive arm angle starts from an initial side of the angle (disc center to arm base) and moves counterclockwise to its terminal side of the angle (disc center to arm tip). A negative arm angle moves clockwise to the terminal side of the angle. The obtained values allow visualizing the initial arm angle in frame 1 and the changes in the other four frames. To find out how much each specimen moved within each time frame, a variable ‘disc displacement’ was calculated by calculating the distance between the disc center position in frame *n* + *1* and frame *n* (Variable_3 in https://github.com/mgm1001/Locomotion_Ophiuroidea.git). Finally, ‘slip angle’ was calculated as the angle between the true disc direction (direction of the disc center movement with respect to the substrate) between frame *n* and *n* + *1* and arm base direction in frame *n*. It was acquired by calculating the angle between the slope of the arm proximal point to the disc center (disc radius) and the slope of the disc position in frames *n* and *n* + *1* with respect to the X-axis (Variable_4 in https://github.com/mgm1001/Locomotion_Ophiuroidea.git). The obtained value represents the direction in which the brittle star moved. The plots of each variable were generated using the matplotlib.pyplot and seaborn libraries. Figure 2 shows a sample result corresponding to each measurement mentioned above. All calculated variables were saved in.csv format for further statistical analysis. The maximum and minimum values in each variable for each species are shown in Tables 1 and 2.

**Table 1:** The maximum and minimum values of sinuosity, arm angle and disc displacement in the three species studied (negative angles represent a clockwise direction)

Species/variables	Sinuosity		Arm angle		Disc displacement		
	(Max)	(Min)	(Max)	(Min)	(Max)	(Min)	(Mean)
*O. superba*	1.40 _A5_	1.0 _A3_	− 37.9 _A5_	− 0.1 _A4_	13.24	1.43	5.22
*O. scolopendrina*	2.52 _A2_	1.0 _A1_	− 81.6 _A5_	0.002 _A5_	11.38	0.16	3.67
				− 0.3*_A4_		2.44*	6.37*
*M. hirsuta*	21.3 _A5_	1.0 _A2_	− 146 _A3_	− 0.2 _A2_	11.24	0.50	4.60

‘A’ stands for ‘arm’, with the corresponding number. The asterisk ‘*’ indicates that the small juveniles *O. scolopendrina* were excluded

**Table 2:** The maximum and minimum slip angle (SA) of arms 1–5 and minimum slip in total arms for 3 species of ophiuroid

Species/SA	Arm1		Arm2		Arm3		Arm4		Arm5		Total
	(Max)	(Min)	(Max)	(Min)	(Max)	(Min)	(Max)	(Min)	(Max)	(Min)	(Min)
*O. superba*	37.8*	0.7	100.6	35.7*	177.4	104.3*	174.4	103.0	141.5	38.4	0.7_A1_
	61.8**			12.4**		101.6**					
*O. scolopendrina*	80.1	1.3	151.4	51.42	179.4	112.1	175.7	94.4	153.5	7.8	1.3_A1_
*M. hirsuta*	58.9	0.8	169.1	34.3	159.6	90.6	176.4	90.0	177.7	45.2	0.8_A1_

The asterisk ‘*’ indicates rowing and ‘**’ indicates reverse rowing movement. The same value was found in total arm minimum slip angle when small juveniles *O. scolopendrina* were included or excluded. ‘A’ stands for ‘arm’, with the corresponding number

### Statistical analysis

The *multivariate_normality* () function from the Pingouin library showed that the data had to be analyzed with a nonparametric PERMANOVA, including all species and all variables (except frames), to test for differences between and within species, and between arms (arms 1–5). Because we needed to assume that individual animals would not necessarily move in a similar way during a single cycle, especially when motor pattern is not fixed, frames at set intervals within a complete cycle could not be considered as a proxy for the same phases in that cycle. One-way ANOVA (Kruskal–Wallis test) was performed using PAST software [23], v.3.22) for all species based on each variable (‘sinuosity’, ‘arm angle’, ‘slip angle’ and ‘disc displacement’) to determine which of the variables were significantly different between species. In addition, as an animal has not moved in Frame 1 and the value of slip angle and disc displacement was zero, we repeated the analysis by excluding Frame 1 (Table 3). For all tests, significance was defined as *P* < 0.05.

**Table 3:** The summary of one-way ANOVA and one-way perMANOVA for inter- and intraspecies comparison, between arms for 3 species of ophiuroid

	*P* value	F value
One-way PerMANOVA		
Interspecies	0.49	0.76
	0.03 (excluding slip angle)	3.54
Intraspecies	0.85 (*M. hirsuta)*	0.51
	0.78 *(O. scolopendrina)*	0.54
	0.98 *(O. superba)*	0.21
Within arms	0.0001	106.3
One way-ANOVA (Kruskal–Wallis)		
Angle	0.03	3.41
Slip angle	0.8	0.22
Disc displacement	2.987E-05	10.71
Sinuosity	8.693E-09	19.51

For One way-ANOVA, the degree of freedom = 2

To check the distribution of the species and the arms in kinematic morphospace, between group principal component analyses (bg-PCA) were done on the correlation matrix of the dataset including all variables associated with all five arms in the five frames for the three species (19 videos in total) using PAST software (v. 3.22). A regular PCA was also done on the correlation matrix of the variation within species using the Sklearn library. Based on a scree plot with a broken stick analysis in the regular PCA, PC1–3 were retained for further interpretation (cumulatively explained 79% of the variation). As the variation explained by PC1-2 and PC1-3 was similar, PC1-3 is not described here. In addition, because of the extensive folding of arms 1 and 5 in two individuals of *M. hirsuta*, they were clearly separated from the morphospace of the other individuals and thus were excluded from the PCA (but were included in other visualizations and tables). For the comparison with the reverse rowing mode in *O. superba* (superba_6), two additional PCAs were run, one with the superba_6 individual (performing the reverse rowing movement) removed from the data and one in which it was included but with arm names modified to reflect the reversed rowing direction: A1:3, A2:4, A3:5, A4:1, A5:2 (i.e., arm 1 changed to arm 3). By this modification (e.g., A4:1), new arm 1 is located on the posterior side opposite to the movement direction, now trailing behind instead of leading. However, this modification does not show any difference in the morphospace (Fig. 5A, B), so the numbering as done for the forward rowing mode could have been retained. As a result, for our analysis we assumed superba_6's arm 1 on the anterior side.

## Results

The substantially longer arms in *M. hirsuta* showed a higher degree of flexibility than the other two species (Fig. 1). The leading arm (arm 1) did not show a specific pattern between the species, and there was no symmetry (the overall curvature shape between two arms on the opposite side of the disc on a specific time frame) in the kinematics of arms 2 and 5 compared to 3 and 4, respectively. Arms 3 and 4 showed the least bending compared with other arms, in both *O. superba* and *O. scolopendrina*.

**Fig. 3:**
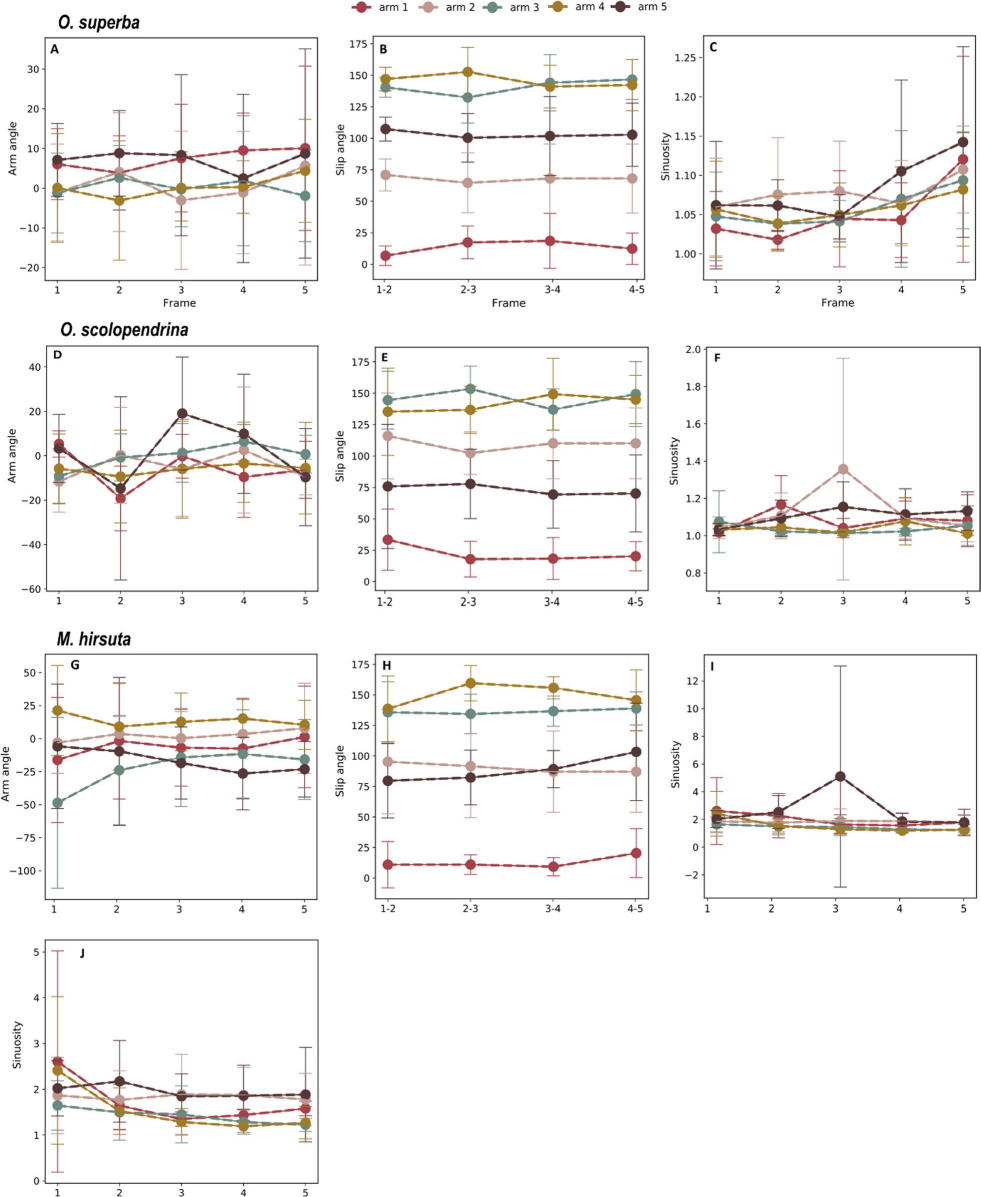
The means and their standard error in arm angle, slip angle, and sinuosity in total trials of *O. superba* (**A**–**C**), *O. scolopendrina* (**D**–**F**), and *M. hirsuta* (**G**–**J**). In plot J, the outliers (extensive folding of arms 1 and 5 in two individuals of *M. hirsuta*) are excluded for a better resolution of the line disparity. The X-axis implies a full cycle of arm movement which was split into four equal intervals, yielding five frames

The variability in the kinematics across all trials for each species (Fig. 3) showed that the arm angle was about zero to + 10 in *O. superba*, about −20 to + 20 in *O. scolopendrina*, and about − 50 to + 25 degrees in *M. hirsuta*. The average-value comparison of slip angle in all three species showed relatively similar values, with arm 1 showing the least and arms 3 and 4 showing the highest values (Fig. 3B, E, H). The slip angle in arm 5 was higher than that of arm 2 in *O. superba*, while it was the opposite in *O. scolopendrina* and fluctuating *in M. hirsuta*. Arm 5 has the highest sinuosity among all species, except for frames 2–3 (arm 2) of *O. superba* and frame 1–3 (arms 1–3) of *O. scolopendrina*, and frame 1 (arms 1 and 4) of *M. hirsuta* (Fig. 3C, F). The peak in frame 3 for *M. hirsuta* (Fig. 3I) is due to extra folding and turning of the arm, where the distal point is located close to the proximal arm. Arm 1 showed the least sinuosity in *O. superba*, unlike the other two species where it is arms 3 and 4 (Fig. 3C, F, I, J).

**Fig. 4:**
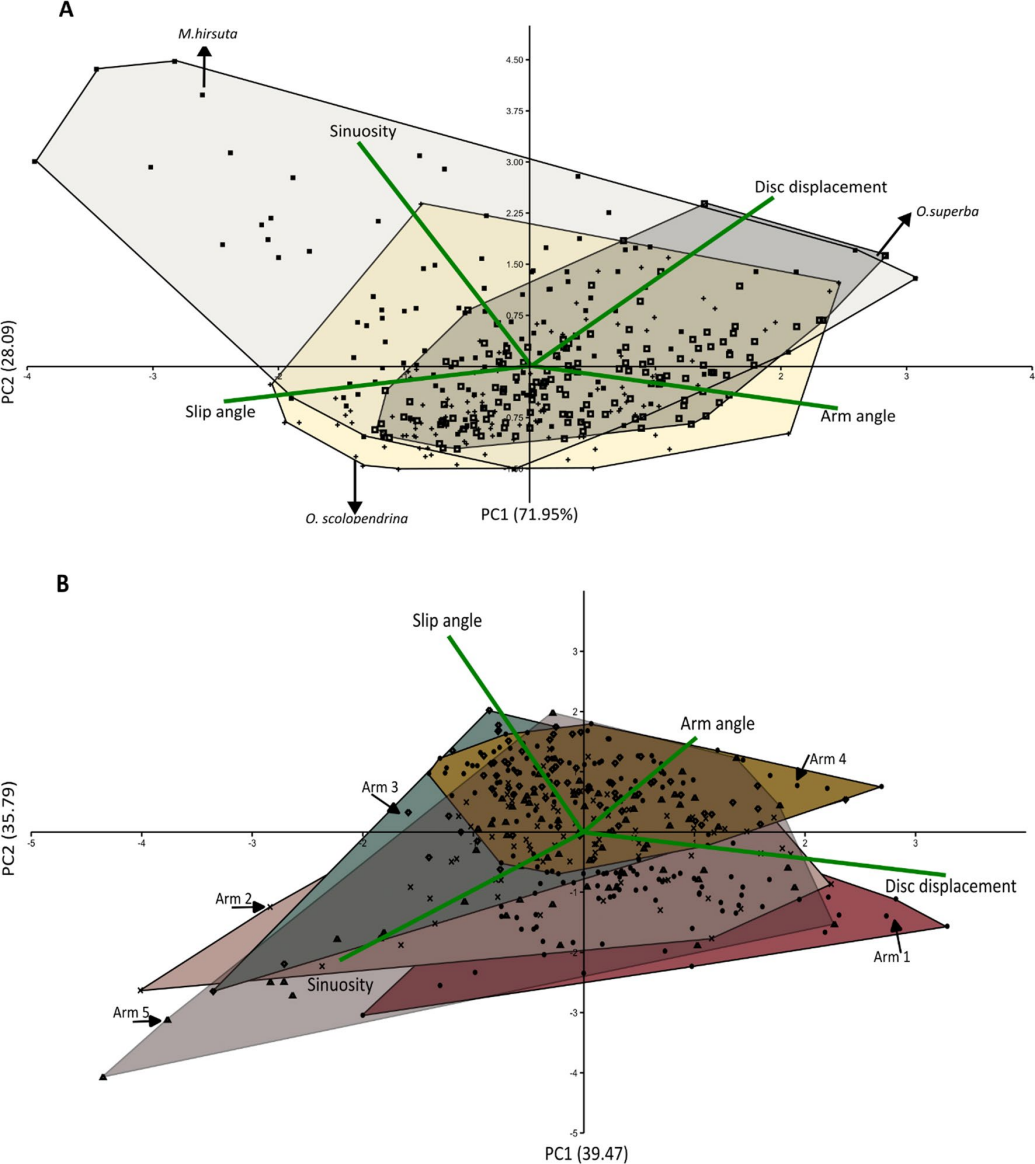
Between-group PCA (PC1–2) for interspecies comparison includes *O. superba*, *O. scolopendrina* and *M. hirsuta* (**A**), and for variability between arms 1–5 (**B**). Since the disc displacement and slip angle in frame 1 was zero, we have removed frame 1 from the data. The colored convex hulls in A, B and C belong to different species, arms and frames categories, respectively. The percentages at labels show the contribution ratios and the lines with variable names represent the loading. For the meaning of "frame" see Fig. 3

The bg-PCA plots show that disc displacement and arm angle have similar loadings on the PC1 for species, and arms but different with respect to PC2 (Fig. 4). Sinuosity and slip angle also have similar magnitude of loading between species and arms in PC1 but different in PC2. Disc displacement and arm angle are positively correlated with PC1 within species and arms (Fig. 4A, B). Disc displacement is negatively correlated with slip angle within species and arms (Fig. 4A, B). However, it is largely independent of sinuosity within species (Fig. 4A) and negatively correlated within arms (Fig. 4B). The sinuosity explains most of the separation of *M. hirsuta* from the other two species (Fig. 4A). Sinuosity is also somehow independent from slip angle and disc displacement, but inversely correlated with arm angle (Fig. 4A). The arms are more distinct with respect to the slip angle, whereas variation within each arm is mostly described by disc displacement, arm angle and sinuosity (Fig. 4B).

**Fig. 5:**
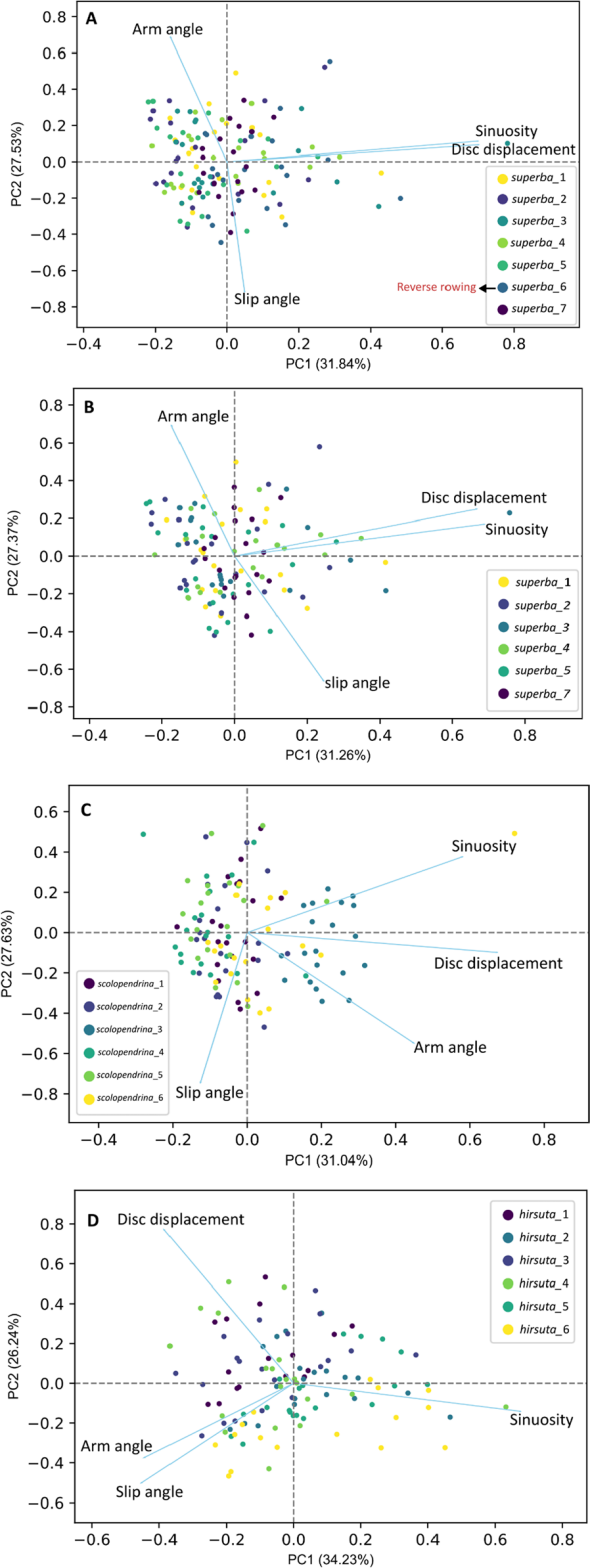
Intraspecific variability in arm mobility traits (PC1–2) for species *O. superba* including all individuals (**A**); without the individual illustrating reverse rowing mode (**B**); *O. scolopendrina* (**C**) and *M. hirsuta* (**D**). The percentages at labels show the contribution ratios and the lines with variable names represent the loading

The PCA on intraspecific variability shows that sinuosity is strongly correlated with disc displacement in *O. superba*, both being independent of arm angle and slip angle (the latter two being strongly inversely correlated with each other) (Fig. 5A, B). Excluding the reverse rowing in *O. superba* (Fig. 5B) did not alter this pattern. Arm angle, sinuosity and disc displacement are all correlated with PC1 in *O. scolopendrina* (Fig. 5C), with individuals scolopendrina_3 and scolopendrina_6 showing the highest PC1 scores. These were the largest individuals, with the other ones being distinctly smaller. This indicates a size dependency of arm kinematics. In *M. hirsuta,* arm angle and slip angle are highly correlated (Fig. 5D) whereas sinuosity is negatively correlated with the other variables.

**Fig. 6:**
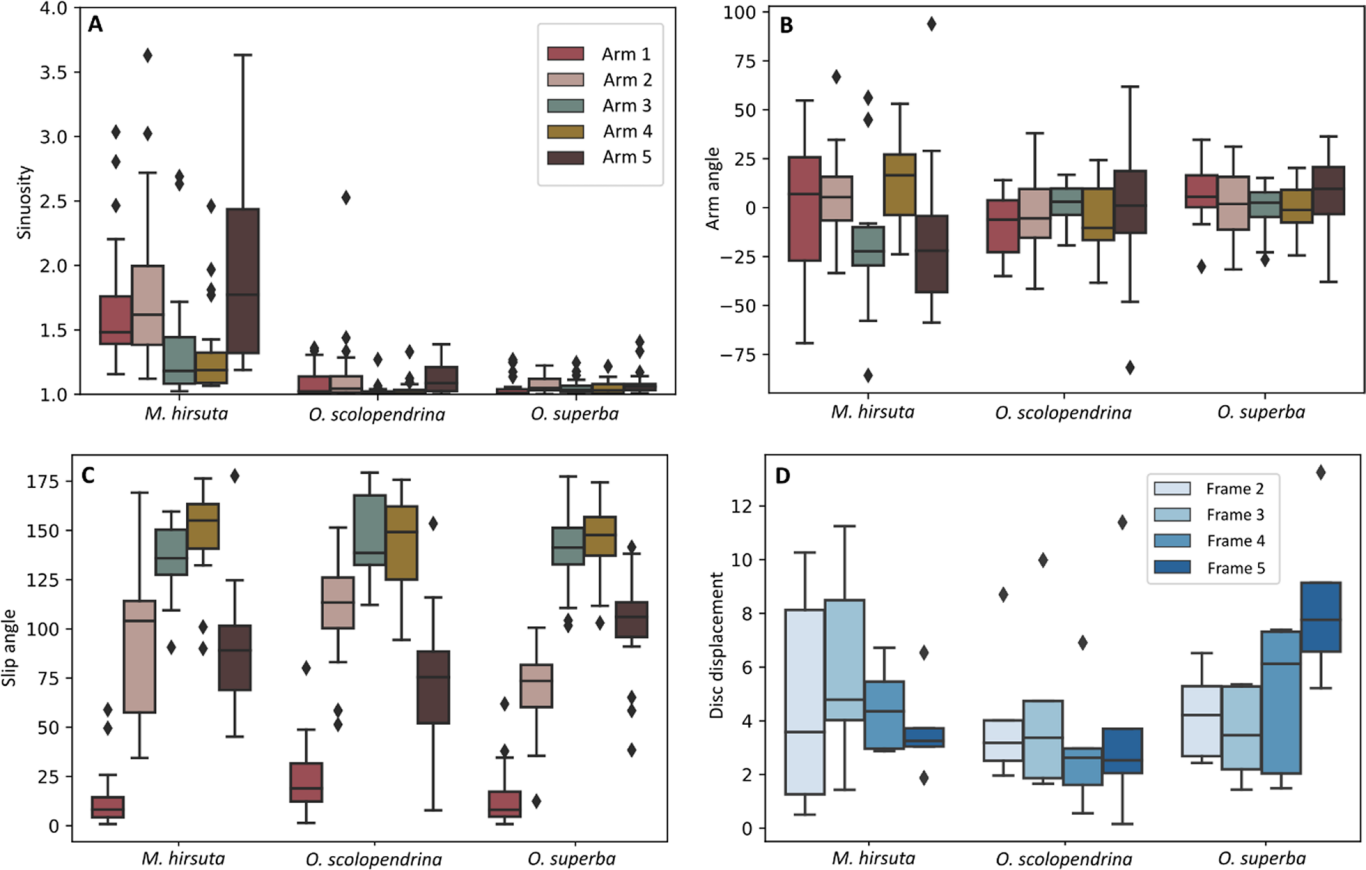
Boxplots of sinuosity (**A**), angle (**B**), slip angle (**C**), and disc displacement (**D**) for *O. superba*, *O. scolopendrina* and *M. hirsuta*, in arms 1–5 and frames 2–5. The boxplots provide the summary of the data as minimum, first quartile, median, third quartile and maximum. The diamond-shaped markers show the outliers. For the meaning of "frame" see Fig. 3

The sinuosity and arm angle showed a wider range of values in *M. hirsuta*, whereas *O. superba* showed the narrowest range (Fig. 6A, B). The highest sinuosity was observed in *M. hirsuta*, much higher than in *O. scolopendrina* and *O. superba* (the latter with the lowest observed value) (Table 1). All three species reached a minimum sinuosity of 1, implying they all at some point fully stretched the arm during a walking cycle. In addition to observing high sinuosity in *M. hirsuta*, a similar interspecific difference was observed for arm angle direction compared with the other two species, which could be correlated with the extensive arm flexibility and wider range of movement in *M. hirsuta* (Table 1 and Figs. 3, 6). After excluding the juveniles of *O. scolopendrina* from the analysis, *O. superba* showed the minimum absolute arm angle among the three species. Arms 2 and 5 in *M. hirsuta* and *O*. *scolopendrina*, respectively, show a wider range of changes for slip angle than other arms and arms 3 and 4 show the highest value in all three species (Fig. 6C). Arm 1 in *O. scolopendrina*, arms 2 and 5 in *M. hirsuta* have the maximum slip angle compared with the corresponding arms in *O*. *superba* (Fig. 6C and Table 2). Within the three species, regardless of the animal size, *O. scolopendrina* showed the least and *M. hirsuta* showed the highest range of variation and also average value in disc displacement, while the highest disc displacement from frame 1–5 is shown in *O. superba* (Fig. 6D and Table 1). As for the disc displacement between species, *O. superba* showed higher values in both maximum and minimum distances. However, there is a bias in disc displacement, because it depends on the size of the animal (cf. scolopendrina_3 and scolopendrina_6 being more separated from the other individuals in Fig. 5C). When using the average disc displacement, excluding the miniature specimens, *M. hirsuta* showed the least displacement.

Due to the biological position of arms on the body and the movement direction, arm one in all species showed the least and arms 3 and 4 showed the highest value in slip angle, and consequently we reported the value for each arm separately to avoid any bias in minimum and maximum angles. The maximum and minimum slip angle among all species corresponding to each arm and the minimum slip angle of the total arms are shown in Table 2. The high slip angle of arm 1 assuming rowing or reverse rowing in *O. superba* was different (Table 2). In general, rowing was the most common locomotory mode compared with reverse rowing mode, accounting for 94.7% of all trials. Arms 2 and 5 in *M. hirsuta* showed the highest slip angle, intermediate in *O. scolopendrina* and finally *O. superba* as the least. All species showed a similar slip angle for arms 3 and 4. The minimum slip angle of *O. superba* and *M. hirsuta* were closer to each other than to *O. scolopendrina*.

The one-way PERMANOVA and one-way ANOVA showed a significant difference (*P* < 0.05) between species for sinuosity, arm angle and disc displacement, but not for inter-individual differences (Table 3). It also shows a significant difference between arms (*P* < 0.05). The Kruskal–Wallis test for all species, but each variable separately, showed significant differences in arm angle (*P* < 0.05), disc displacement (*P* < 0.05) and sinuosity (*P* < 0.05), but an insignificant difference in slip angle.

## Discussion

Our hypothesis on a difference in kinematics between the three species *O. superba*, *O. scolopendrina*, and *M. hirsuta* was supported by the significant differences for the variables arm angle, sinuosity and disc displacement. In addition to LeClair and LaBarbera [24], who also reported significant interspecies differences in mean maximal intersegmental rotations in locomotion, our result showed significant differences in horizontal arm motions (e.g., arm angle). Although they found no functional implications in their result, we found that the significant interspecific differences in our study do describe variation in arm flexibility (Table 1). Quantifying sinuosity for each complete arm allows to identify differences in locomotion better than only using the maximum inter-plate rotations along the arm, which was already studied by LeClair and LaBarbera [24]. This makes sense considering that a similar arm plate angle rotation can correspond to multiple bending patterns in an arm.

The sinuosity analysis showed that for each species, the side arms (arms 2 and 5) and then the leading arm (arms 1) tend to bend most during locomotion, whereas the posterior arms (arms 3 and 4) show less bending and thus act more as trailing arms (Figs. 5 and 6). The long-armed species, *M. hirsuta,* shows the highest flexibility, being able to bend and shorten the arm extension from the disc by approximately 20 × and exhibit an arm motion up to 146° (Table 1 and Fig. 6). We also found that *O. superba*, presenting the lowest sinuosity*,* is more rigid than *O. scolopendrina*. In *O. superba* a maximum arm angle of 37.9° by one of the front arms (arm 5) was observed, while *O. scolopendrina* showed a maximum arm angle of 81.6 ^o^ for the same arm (Table 1 and Fig. 6). In *O. scolopendrina*, juveniles showed smaller arms angles, whereas adults showed larger, suggesting some level of size-dependency of arm angle. This should be studied further with individuals in a wider size range.

Slip angle was analyzed in order to provide more information on the direction of the animal moving with respect to disc rotation. Higher slip angles in arm 5 than in arm 2 in *O. superba* suggest that individuals on average tended to move to the right within all trials, while those of *O. scolopendrina* moved to the left (lower slip angles in arm 5 than arm 2) (Fig. 3B,E). In general, arms 3 and 4 are moving in the opposite direction of the moving direction, confirming a bilaterally coordinated movement (Table 2) [5]. The slip angle in the side arms to the movement direction (arms 2 and 5) are also more similar to each other, however, arm 5 showed more angle variations and thus seemed more active than arm 2 (Tables 1, 2 and Fig. 6).

The vertical arm movements, which might have important roles in animal locomotion, were not measured in this study. The mean anterior arm angles (arms 2 and 5) across species are 88° ± 32.9 and 89° ± 29.9 to the forward direction, respectively, which is in line with Clark [6] stating that they are perpendicular to the movement direction. In addition, the interspecies mean of posterior arm angles were 141° ± 18.3 and 145° ± 20.9 (arms 3 and 4), respectively, indicating that they were moving in mirror with respect to each other. Arm 1, considered to be the leading arm, has a mean slip angle of 16° ± 15.9. Our behavioral observation indicated that arm 1 has a leading role, but also the posterior arms (arms 3 and 4) exhibit horizontal movements that suggest being passively dragged in a mirrored manner whereas the front arms (arms 2 and 5) show more paddling-like movements accompanied with vertical movements to help displace the disc. This is in agreement with Clark [6]. The leading arm and the next one (arm 2) showed a wider range of arm angle changes during reverse rowing (Table 2). It implies that both arms in reverse rowing have important roles in moving forward. Our results of slip angle showed that the animal chooses rowing mode for moving into the direction of either of the arms (that arm is assigned as arm 1/leading arm). Additionally, we found that moving in a direction between two arms would make the animal switch to reverse rowing mode. In this mode, two arms would play active roles in locomotion (the arm located in the opposite direction of movement is labeled as arm 1 but is now the trailing arm). Disc displacement showed to be positively correlated with arm angle and negatively with slip angle within species and arm (Fig. 4A,B). This suggests that the arms move forward within the direction of body movement and hence move the disc to a new location. Average displacement was highest in *O. superba* (Fig. 6), despite lower arm flexibility. Whether or not a stronger musculoskeletal system could explain this needs to be investigated. Additionally, the effect of disc displacement should be analyzed by removing the size effect within the trials.

Our study showed that the sinuosity, disc displacement, and arm angle, respectively, are the most important parameters compared with slip angle to interpret animal locomotion as the species were significantly different regarding these variables (Table 3 and Figs. 4, 5, 6). However, slip angle could also illuminate other perspectives, such as how the animal changes the anterior directions and how the body moves the disc unidirectionally.

In the study of Litvinova [20], it was hypothesized that comb-shaped vertebrae allow the arms to bend in a horizontal direction, while the universal type would allow bending in every direction (but not twisting, like in prehensile species). This is in agreement with our behavioral observations, although we did observe some vertical motion of the front arms in *O. scolopendrina* and *O. superba* (but was not quantified in this study). In addition, it was suggested that the extended keel in some ophiuroid species, such as *M. hirsuta* in our study*,* may play a role in the arm movements [24, 25]. The high sinuosity of this taxon could be explained by the presence of such an extended keel, as in such vertebrae the central projection is reduced, there is a large proximal depression and extended structure on the dorso-distal face, and there are accessory aboral muscles between the proximal depression and distal extension [3, 17]. Thus, among the zygospondylous species of the current study, keeled vertebrae could play a prominent role in arm flexibility.

This study did not allow to perform a quantitative comparative analysis on reverse rowing in all three species*,* as it was recorded only once in *O. superba*. A controlled experiment, as done with *O. superba*, could capture to what degree this pattern simply reflects a mirrored kinematics in arm use, or whether a more versatile, underlying motor control is active. Also, the aspect of size dependency of the arm kinematics needs further attention, because when the miniature *O. scolopendrina* was included, the maximum and minimum values of some studied variables (e.g., arm angle) differed between species. In addition, the number of vertebrae accompanied with their size should be considered too, because arms with similar length but different number of vertebrae may show different kinematic behavior. Overall locomotory performance traits could also incorporate speed in relation to arm morphology and animal size. Extending this work to a larger number of species within a phylogenetic context could elucidate the evolutionary pattern behind structural and functional adaptations in brittle star locomotion. Despite ongoing interest in locomotion modeling, Kano et al. [10, 11] concluded that the biological basis of brittle stars is still lacking. Modeling of arm movement could help design autonomous robots to determine their moving directions and how to move as effectively as the brittle stars [12]. Further, including artificial neural network (ANN) analysis to compare observed with predicted kinematic output might be further help in robotics research.

## Conclusion

The three species, as case studies, contributed to a methodological exploration of new variables and ways to summarize arm kinematics graphically. It showed that the sinuosity, disc displacement and arm angle are the most important parameters compared with slip angle to interpret locomotion in brittle stars. We found that rowing mode occurs more frequently than reverse rowing mode in brittle star locomotion. The result illustrated that arm 1 has a leading role, the posterior arms (arms 3 and 4) were pulled or trailing in a mirrored manner, whereas the front arms (arms 2 and 5) show more paddling-like movements, accompanied by vertical movements to help displace the disc. However, the muscle activity and vertical movements need to be explored to assess the arm activeness versus passiveness. We found that the extended keel in *M. hirsuta* may play a role in the arm movements and could explain one aspect of its higher flexibility compared to *O. scolopendrina* and *O. superba*. The length of the arm angle was also important in the range of the arm angle, as *M. hirsuta* with the longest and miniature *O. scolopendrina* with the shortest arms, showed the highest and lowest arm angles among the studied species, respectively. Interestingly, the most rigid species of the current study (*O. superba*) showed the most disc displacement within frames, and *M. hirsuta* and *O. scolopendrina* showed rather flexible bodies. In this regard, either high-speed rigidness or flexibility could benefit robotic designs. We believe that the methods of the current study can be extended to all five-armed ophiuroids for further research and can offer a substantial contribution to robotics.

## Supplementary Information

**Additional file 1.** Morphological comparison of external and internal characters in three ophiuroid families derived from the DELTA interactive key by Goharimanesh et al. [17]. OP: Oral plate, DP: Dental plate.

## Data Availability

The Python scripts developed in this study are available in GitHub (https://github.com/mgm1001/Locomotion_Ophiuroidea.git). The datasets used during the current study are available from the corresponding author on reasonable request.

## References

[1] Stöhr S, Schierwater B, Desalle R (2021). Phylum echinodermata. Invertebrate zoology: a tree of life approach.

[2] Lal SP, Yamada K. Evolutionary distributed control of a biologically inspired modular robot. Frontiers in Evolutionary Robotics. 2008. IntechOpen.com. 10.5772/5472

[3] LeClair E (1996). Arm joint articulations in the ophiuran brittlestars (Echinodermata: Ophiuroidea): a morphometric analysis of ontogenetic, serial, and interspecific variation. J Zool.

[4] Lawrence JM. Echinodermata. TJ Pandian and FJ Vernberg (eds.). Animal Energetics, vol. 2. Bivalvia through Reptilia. Academic Press, San Diego. 1987;229–307.

[5] Astley HC (2012). Getting around when you’re round: quantitative analysis of the locomotion of the blunt-spined brittle star, *Ophiocoma echinata*. J Exp Biol.

[6] Clark EG (2019). Ophiuroid locomotion from fundamental structures to integrated systems. Zoosymposia.

[7] Watanabe W, Kano T, Suzuki S, Ishiguro A (2011). A decentralized control scheme for orchestrating versatile arm movements in ophiuroid omnidirectional locomotion. J R Soc Interface.

[8] Kano T, Sato E, Ono T, Aonuma H, Matsuzaka Y, Ishiguro A. A brittle star-like robot capable of immediately adapting to unexpected physical damage. R Soc Open Sci. 2017;4:171200. 10.1098/rsos.171200 10.1098/rsos.171200 PMC5750017 29308250

[9] Zueva O, Khoury M, Heinzeller T, Mashanova D, Mashanov V (2018). The complex simplicity of the brittle star nervous system. Front Zool.

[10] Kano T, Kanauchi D, Ono T, Aonuma H, Ishiguro A (2019). Flexible coordination of flexible limbs: decentralized control scheme for inter-and intra-limb coordination in brittle stars' locomotion. Front Neurorobot.

[11] Kano T, Kanauchi D, Aonuma H, Clark EG, Ishiguro A (2019). Decentralized control mechanism for determination of moving direction in brittle stars with penta-radially symmetric body. Front Neurorobot.

[12] Patterson ZJ, Sabelhaus AP, Chin K, Hellebrekers T, Majidi C. An untethered brittle star-inspired soft robot for closed-loop underwater locomotion. In 2020 IEEE/RSJ International Conference on Intelligent Robots and Systems (IROS). 2020;8758–8764.

[13] Matsuzaka Y, Sato E, Kano T, Aonuma H, Ishiguro A (2017). Non-centralized and functionally localized nervous system of ophiuroids: evidence from topical anesthetic experiments. Biol Open.

[14] Wakita D, Kagaya K, Aonuma H (2020). A general model of locomotion of brittle stars with a variable number of arms. J R Soc Interface.

[15] Goharimanesh M, Ghassemzadeh F, De Kegel B (2022). The evolutionary relationship between arm vertebrae shape and ecological lifestyle in brittle stars (Echinodermata: Ophiuroidea). J Anat.

[16] Stöhr S, O’Hara T, Thuy B, eds. World Ophiuroidea Database. 2022. Available from http://www.marinespecies.org/ophiuroidea. Accessed 04 Oct 2022.

[17] Goharimanesh M, Stöhr S, Mirshamsi O, Ghassemzadeh F, Adriaens D (2021). Interactive identification key to all brittle star families (Echinodermata; Ophiuroidea) leads to revised morphological descriptions. Eur J Taxon.

[18] Stöhr S, Clark EG, Thuy B, Darroch SA (2019). Comparison of 2D SEM imaging with 3D micro-tomographic imaging for phylogenetic inference in brittle stars (Echinodermata: Ophiuroidea). Zoosymposia.

[19] Goharimanesh M, Mirshamsi O, Stöhr S, Ghassemzadeh F, Adriaens D (2021). New data on brittle stars (Echinodermata: Ophiuroidea) from the Persian Gulf and Oman Sea, Iran. IJAB.

[20] Litvinova NM, David B, Guille A, Féral J-P, Roux M (1994). The life forms of Ophiuroidea (based on the morphological structures of their arms). Echinoderms through time.

[21] Mary H, Brouhard GJ. Kappa (κ): analysis of curvature in biological image data using B-splines. BioRxiv. 2019. 852772. doi: 10.1101/852772

[22] Leucht PM. The directional dynamics of the commercial tractor-semitrailer vehicle during braking. SAE trans. 1970;1146–1156.

[23] Hammer Ø, Harper DAT, Ryan PD (2001). Past: Paleontological statics software package for education and data analysis. Palaeontol.

[24] LeClair EE, LaBarbera MC (1997). An in vivo comparative study of intersegmental flexibility in the ophiuroid arm. Biol Bull.

[25] Clark EG, Hutchinson JR, Darroch SA (2018). Integrating morphology and in vivo skeletal mobility with digital models to infer function in brittle star arms. J Anat.

