# Supplementary material for: A methodological exploration to study 2D arm kinematics in Ophiuroidea (Echinodermata)
Source: Front Zool. 2023 Apr 21;20:15. doi: 10.1186/s12983-023-00495-y (PMC10120178; doi:10.1186/s12983-023-00495-y)
Supplement: Supplementary file 1 — Additional file 1. Morphological comparison of external and internal characters in three ophiuroid families derived from the DELTA interactive key by Goharimanesh et al. [17]. OP: Oral plate, DP: Dental plate. [file 12983_2023_495_MOESM1_ESM.docx]

Supplementary File 1. Morphological comparison of external and internal characters in three ophiuroid families derived from the DELTA interactive key by Goharimanesh et al. (2021b). OP: Oral plate, DP: Dental plate.

| **Characteristics** | **Ophiocomidae**  (e.g., *Ophiocoma scolopendrina*) | **Ophiolepididae**  (e.g., *Ophiolepis superba*) | **Ophiotrichidae**  (e.g., *Macrophiothrix hirsuta*) |
| --- | --- | --- | --- |
| **Arm** | length 3–4x disc diameter | length 3x disc diameter | length more than 4x disc diameter |
| **Accessory dorsal arm plate** | absent | present | absent |
| **Accessory ventral arm plate** | absent | present; or absent | absent |
| **Arm spine cross section** | oval | round | round |
| **The ratio of thickness of distal arm spine to maximum thickness (in a given cross-sectional plane)** | 0.6–0.9; or 0.9–1 | 0.6–0.9 | 0.6–0.9 |
| **Arm spines** | flat; or cylindrical; or pointed | pointed | pointed |
| **Arm spines** | predominantly erect, standing perpendicular to arm axis | predominantly parallel to arm axis (appressed) | predominantly erect, standing perpendicular to arm axis |
| **Longest arm spines** | longer than two segments | shorter than half a segment | longer than two segments |
| **Tentacle scales** | nearly as long as wide | slightly longer than wide | more than two times as long as wide |
| **Tentacle scales** | shorter than nearest spine | shorter than nearest spine | as long as nearest spine |
| **Tentacle scales** | cross section oval | cross section oval | cross section round |
| **Arm spine articulation** | with a fully developed sigmoidal fold | without sigmoidal fold | without sigmoidal fold |
| **Arm spine articulation** | dorsalwards increasing in size | larger in middle | larger in middle |
| **Nerve opening** | smaller than muscle opening | approximately as large as muscle opening | approximately as large as muscle opening |
| **Disc** | pentagonal; or circular | pentagonal; or circular | circular |
| **Integument** | obscuring plates/scales | not obscuring plates/scales | not obscuring plates/scales |
| **Dorsal disc** | with granules | without granules | without granules |
| **Dorsal disc** | with concealed primary plates | with obvious primary plates | with concealed primary plates |
| **Dorsal disc** | With or without spines | without spines | with spines |
| **Radial shield** | with granules | without granules/spines | with spines |
| **Radial shield** | length less than one third of the disc radius | length between one third and half of the disc radius | length more than half of the disc radius |
| **Radial shield** | scalene (oblique) triangular | isosceles (mirror-symmetric) triangular to pear-shaped | scalene (oblique) triangular |
| **Ventral interradii** | with granules | without granules/spines | with spines |
| **Ventral teeth** | several (tooth papillae) | single | several (tooth papillae) |
| **Infradental papillae on oral plate** | present | present | absent |
| **Buccal scales on OP** | absent | present | present |
| **Lateral oral papillae on OP** | present; or absent | absent | absent |
| **Adoral shield spine** | present | present | absent |
| **Lyman's ossicle** | present | present | absent |
| **Oral shield** | about as long as wide | much longer than wide | much wider than long |
| **Adoral shield** | separated | meeting in front of the oral shield | separated |
| **Dental plate** | equal width all over | equal width all over; or dorsal half widest | ventral half widest |
| **Dental sockets** | circular; or heart-shaped | circular; or heart-shaped | slit-shaped; or circular |
| **Dental sockets** | equal to or more than 50% of the width | equal to or more than 50% of the width | less than 50% of the width; or equal to or more than 50% of the width |
| **At least one socket on DP as** | perforation with septum | depression or perforation without septum | perforation with septum |
| **Teeth** | block-like (with square tip) | block-like (with square tip) | flat (with round or slightly pointed tip, but never spine-like) |
| **Teeth on DP arranged as** | a single column on half of dental plate and other half with a cluster of tooth papillae (ventral cluster); or a single column on 2/3 and a cluster of tooth papillae on 1/3 of dental plate | single column | a single column on half of dental plate and other half with a cluster of tooth papillae (ventral cluster) |
| **Vertebrae beyond segment 5** | with short keel | with short keel | with extended keel |

38 differences.
